# Reduction in Wearable-Derived Maximal Oxygen Consumption (VO₂max) During Tamsulosin Use in an Endurance Athlete

**DOI:** 10.7759/cureus.109685

**Published:** 2026-05-26

**Authors:** Hunter Cohn, Ava Zamani, Michael Sessine, Michael L Cher

**Affiliations:** 1 Medicine, Wayne State University School of Medicine, Detroit, USA; 2 Urology, Wayne State University, Detroit, USA

**Keywords:** adverse drug effects, alpha-1 adrenergic antagonists, benign prostatic hyperplasia, exercise tolerance, maximal oxygen consumption, tamsulosin, wearable devices

## Abstract

α₁-adrenergic antagonists such as tamsulosin are widely prescribed for lower urinary tract symptoms (LUTS) secondary to benign prostatic hyperplasia. While their systemic vascular effects are well recognized, their impact on aerobic performance remains unclear. We describe a case of a competitive endurance athlete who observed a decline in wearable-derived maximal oxygen consumption (VO₂max) temporally associated with tamsulosin therapy.

A male endurance runner with LUTS was initiated on treatment with tamsulosin. Subsequently, he noted an increased perceived level of exertion during training and a reduction in estimated VO₂max during activity as recorded by his smartwatch. Longitudinal running data from his device were reviewed across three consecutive time periods: prior to initiation of tamsulosin (from August 15, 2022, to March 1, 2023), during daily tamsulosin therapy (from March 1, 2023, to February 7, 2024), and following transition to daily tadalafil therapy (from February 7, 2024, to June 15, 2024). A total of 113 outdoor running sessions were included, demonstrating a decline in estimated VO₂max from baseline pre-tamsulosin (48.19 mL/kg/minute) to daily tamsulosin use (45.68 mL/kg/minute) (p = 6.42 × 10^-11^), with partial recovery after initiation of tadalafil (46.21 mL/kg/minute) (p = 6.77 × 10^-5^). This pattern was also observed among runs performed at similar paces.

Although causality cannot be established from a single observation, this case suggests that α₁-adrenergic blockade may influence aerobic performance. Further investigation in larger cohorts and controlled physiologic studies is warranted to better characterize this relationship and its potential impact on exercise capacity.

## Introduction

α₁-adrenergic antagonists are widely prescribed as first-line pharmacologic therapy for lower urinary tract symptoms (LUTS) secondary to benign prostatic hyperplasia (BPH) [1]. Tamsulosin is a selective α₁-adrenergic receptor antagonist that improves urinary flow by relaxing smooth muscle in the prostate and bladder neck [2,3]. Although relatively uroselective, adverse effects such as orthostatic hypotension, dizziness, and fatigue are well documented and are attributed to peripheral vasodilation and reduced sympathetic tone resulting from α₁-adrenergic blockage [1]. While these hemodynamic effects are generally mild, they reflect systemic vascular changes that may become more pronounced during physiologic stress, such as exercise.

Maximal oxygen consumption (VO₂max) represents the maximal rate of oxygen uptake by skeletal muscle during exercise and reflects the integrated function of cardiovascular output, pulmonary gas exchange, and peripheral oxygen utilization [4,5]. It can be expressed in liters per minute (L/minute) of oxygen or in milliliters per kilogram per minute (mL/kg/minute) relative to body mass, making it a widely used measure of aerobic capacity and cardiorespiratory fitness [4,5]. Factors that influence VO₂max include body fat percentage, age, sex, and fitness level, with regular physical activity and exercise leading to greater VO₂max [6-8]. Higher VO₂max is associated with greater aerobic fitness, endurance, and athletic performance [9,10]. Accordingly, even modest changes in VO₂max may meaningfully affect exercise performance in trained individuals; the minimum clinically important difference has been estimated at 1.5-3.6 mL/kg/minute depending on population and methodology [4,11-13].

In recent years, wearable devices, including select smartwatches, have enabled individual longitudinal tracking of estimated VO₂max using relationships between heart rate and pace during exercise [14]. These technologies provide continuous physiologic monitoring that may reveal subtle changes in performance associated with training, illness, or medication exposure.

Although the systemic vascular effects of α₁-adrenergic blockade are well documented, their potential influence on aerobic performance and VO₂max levels has not been well studied. Herein, we describe a case of a competitive endurance athlete who observed a reduction in wearable-derived VO₂max temporally associated with tamsulosin therapy.

## Case presentation

A 61-year-old male endurance athlete with a history of LUTS attributed to BPH presented for evaluation and management of progressively bothersome urinary symptoms. Medical history included hypertension controlled with lisinopril and hypercholesterolemia managed with simvastatin. He had no prior surgical history. Family history was notable for lung cancer. He reported consuming roughly one to two alcoholic beverages once to twice a week and denied any history of smoking or other illicit drug use.

The patient had been an avid runner and cyclist for several decades and regularly participated in competitive road races ranging from 5 km to half-marathons. As part of his training, he routinely monitored performance metrics using a Garmin Forerunner 945 smartwatch (Garmin, Olathe, KS), which estimated VO₂max using Firstbeat physiological analytics derived from wrist-based optical heart rate measurements and pace data collected during outdoor exercise.

He started tamsulosin therapy on August 13, 2022, which improved his urinary symptoms. However, he subsequently developed intermittent dizziness upon standing and perceived that running required greater exertion than typically expected for a given pace, both during outdoor runs and on the indoor treadmill. These symptoms appeared more pronounced in warmer conditions or with direct sun exposure.

Upon reviewing his longitudinal performance metrics recorded by his wearable device, he noted a decline in his estimated VO₂max following initiation of tamsulosin. Given these observations, tamsulosin was discontinued, and tadalafil was instead initiated to manage LUTS. Following this medication switch, the patient reported that running felt subjectively easier, although urinary symptom control was less robust. He eventually chose surgical therapy for his prostate enlargement.

The patient shared these observations and provided a deidentified spreadsheet of VO₂max data collected from his wearable device over this period. With his permission, we reviewed the smartwatch-derived data recorded during exercise.

The device provides VO₂max estimates only during outdoor activity using heart rate and pace. As the patient’s only outdoor activity during this period was running, all data were derived from running. These data included date, distance, heart rate, pace, cadence, elevation, and environmental conditions recorded during each run.

Data were examined across three consecutive time periods: at baseline, prior to initiation of tamsulosin therapy (from August 13, 2022, to March 1, 2023), during daily tamsulosin use (from March 1, 2023, to February 7, 2024), and following transition to daily tadalafil (from February 7, 2024, to June 15, 2024).

To account for the influence of exercise intensity on VO₂max estimates, trends were also evaluated among runs performed at similar paces (8.5 and 9.5 minutes per mile). A total of 113 outdoor running sessions with VO₂max estimates were recorded during this period, including 31 runs during the pre-tamsulosin period, 54 during daily tamsulosin use, and 28 following transition to daily tadalafil.

VO₂max was highest prior to initiation of tamsulosin therapy, with values averaging 48.19 mL/kg/minute. During daily tamsulosin use, VO₂max was consistently lower at 45.68 mL/kg/minute. Following discontinuation of tamsulosin and initiation of tadalafil, VO₂max partially recovered to 46.21 mL/kg/minute, although it remained below baseline (pre-tamsulosin) levels. These observations are summarized in Table 1.

**Table 1:** VO₂max estimates across medication periods for all recorded runs Overall differences in VO₂max across medication periods were evaluated using one-way analysis of variance, followed by Tukey-Kramer post hoc testing for pairwise comparisons to account for unequal sample sizes. Pairwise comparisons included NoTx vs. TamTx, TamTx vs. TadaTx, and NoTx vs. TadaTx NoTx: no tamsulosin period; TamTx: daily tamsulosin period; TadaTx: tadalafil period

Time period	Average VO_2_max	Standard deviation VO_2_max	Number of runs	Average run distance (miles)	Average minutes per mile	Q-statistic for VO_2_max comparison	p values for VO_2_max comparison	
NoTx (from August 13, 2022, to March 1, 2023)	48.19	0.60	31	5.45	8.82	26.37	NoTx vs. TamTx: p = 6.42 × 10^-11^	
								TamTx (from March 1, 2023, to February 7, 2024)	45.68	0.47	54	5.91	9.49	1.61	TamTx vs. TadaTx: p = 6.42 × 10^-11^	
TadaTx (from February 7, 2024, to June 15, 2024)	46.21	0.78	28	5.24	9.20	0.85	NoTx vs. TadaTx: p = 6.77 × 10^-5^									

**Figure 1:**
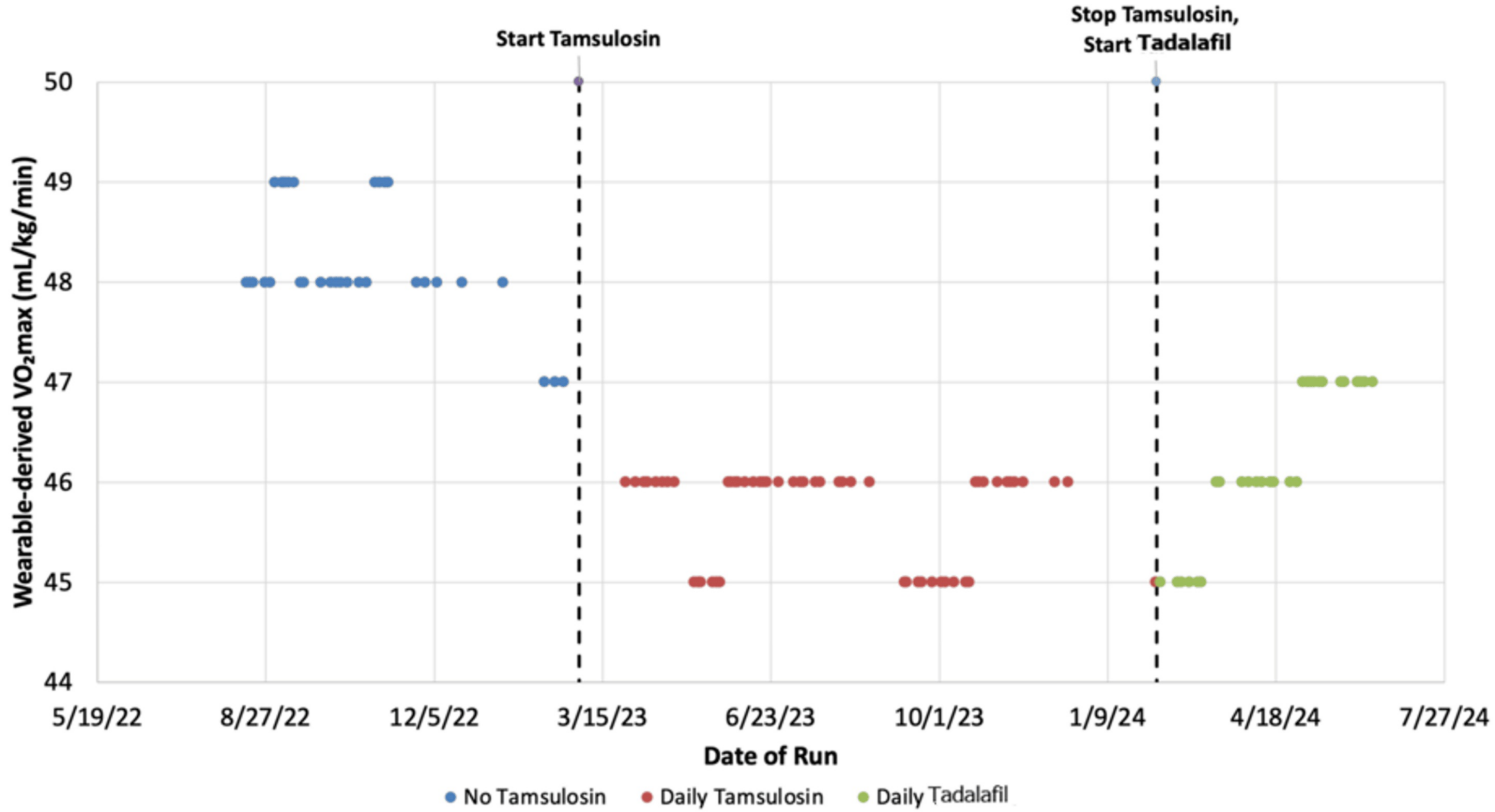
Longitudinal wearable-derived VO₂max estimates across medication periods Vertical dashed lines indicate initiation of tamsulosin therapy and subsequent discontinuation of tamsulosin and transition to tadalafil. Each point represents a single run

Overall, the temporal pattern of VO₂max across the three medication periods showed a decline from baseline after initiation of tamsulosin therapy, with partial recovery after the transition to tadalafil, as illustrated in Figure 1.

This pattern persisted among runs performed at comparable pacing ranges of 8.5-9.5 minutes per mile, as detailed in Table 2.

**Table 2:** VO₂max estimates across medication periods for only runs performed at a pace of 8.5-9.5 minutes per mile The pace-restricted analysis included only runs performed at similar paces to control for exercise intensity. Overall differences in VO₂max across medication periods were evaluated using one-way analysis of variance, followed by Tukey-Kramer post hoc testing for pairwise comparisons to account for unequal sample sizes. Pairwise comparisons included NoTx vs. TamTx, TamTx vs. TadaTx, and NoTx vs. TadaTx NoTx: no tamsulosin period; TamTx: daily tamsulosin period; TadaTx: tadalafil period

Time period	Average VO_2_max	Standard deviation VO_2_max	Number of runs	Average run distance (miles)	Average minutes per mile	Q-statistic for VO_2_max comparison	p values for VO_2_max comparison	
NoTx (from August 13, 2022, to March 1, 2023)	48.09	0.53	21	5.39	8.97	21.05	NoTx vs. TamTx: p = 1.33 × 10^-13^	
								TamTx (from March 1, 2023, to February 7, 2024)	45.66	0.48	32	5.16	9.06	13.15	TamTx vs. TadaTx: p = 1.27 × 10^-9^	
TadaTx (from February 7, 2024, to June 15, 2024)	46.40	0.75	20	5.13	8.99	6.32	TadaTx vs. NoTx: p = 8.59 × 10^-6^									

## Discussion

We present a case of a competitive endurance athlete in whom changes in estimated VO₂max during running were observed following initiation of tamsulosin therapy for LUTS. A review of longitudinal smartwatch-derived data showed lower mean VO₂max during daily tamsulosin use compared with baseline, with partial recovery after transitioning to tadalafil. This pattern persisted with restriction to similar paces. The consistent association between tamsulosin exposure and reduced estimated VO₂max in this athlete suggests that the medication may have measurable effects on aerobic performance.

Blockage of α₁-adrenergic receptors may alter peripheral vascular tone and blood pressure regulation during exertion, potentially influencing cardiovascular responses to endurance exercise [15,16]. In physically active individuals, even modest changes in vascular resistance or perfusion dynamics could increase perceived exertion or reduce exercise efficiency. In this case, the patient reported intermittent orthostatic symptoms and greater perceived running effort while taking tamsulosin regularly. While the precise physiologic mechanism cannot be determined from a single observation, the patient’s symptoms, together with the known systemic hemodynamic effects of tamsulosin, provide a plausible context for the decline in estimated VO₂max.

Several pharmacologic considerations may further explain this association. Tamsulosin is extensively metabolized by CYP3A4 and CYP2D6, and interindividual differences in CYP2D6 activity may influence drug plasma concentrations and the magnitude of systemic hemodynamic effects [17]. Emerging evidence has also linked tamsulosin to rare cases of drug-induced interstitial lung disease, particularly in individuals with reduced CYP2D6 metabolism [18]. Although there was no clinical evidence of ILD or other pulmonary pathology in this patient, these reports highlight the potential for variability in systemic responses to tamsulosin exposure. Additionally, reactive drug metabolites have been implicated in oxidative stress and immune-mediated tissue injury, mechanisms increasingly recognized in drug-induced pulmonary toxicity [18,19]. While CYP2D6 status was not evaluated in this patient and these proposed mechanisms remain speculative, they may warrant further investigation in future studies examining physiologic responses to α₁-adrenergic blockade. Overall, the patient’s reported orthostatic symptoms and increased perceived exertion are still plausibly explained by the known cardiovascular and hemodynamic effects of α₁-adrenergic blockade.

Importantly, tadalafil itself has systemic vasodilatory and cardiopulmonary effects that may influence exercise performance; therefore, the observed partial recovery in estimated VO₂max may reflect an active physiologic effect of tadalafil rather than solely the reversal of tamsulosin exposure. Notably, despite this partial recovery, estimated VO₂max values did not return to baseline, and the patient ultimately pursued procedural management of his LUTS in an effort to return closer to his prior exercise baseline. Although this observation is limited to a single endurance athlete, it raises broader questions regarding whether α₁-adrenergic antagonists may influence perceived fitness, exercise tolerance, or overall physical performance in broader patient populations.

This report has several limitations. It describes a single patient; thus, causality cannot be established. Wearable-derived VO₂max is an estimate rather than a direct physiologic measure and may be influenced by environmental factors, illness, pulmonary and cardiovascular physiologic variation, and changes in training patterns over time. Relatedly, the number of recorded runs differed across medication periods, although each period included multiple longitudinal observations over time, and variation in training volume or conditioning may have contributed to differences in estimated VO₂max. Additionally, heart rate data were obtained using the smartwatch’s wrist-based optical sensor rather than a chest strap monitor. Optical heart rate sensors may be susceptible to motion artifact during running, including cadence-related inaccuracies, which could introduce variability into wearable-derived VO₂max estimates. Environmental conditions such as heat and humidity were not controlled for in the analysis. Because wearable-derived VO₂max estimates rely in part on heart rate responses during exercise, seasonal variation in temperature and environmental stress may have influenced measured values and represent a potential confounder. Furthermore, the observational period spanned nearly two years in a patient in his sixth decade of life, and age-related declines in aerobic capacity over time may have contributed to the incomplete return of estimated VO₂max to baseline levels following transition to tadalafil. Despite these limitations, the longitudinal data in this case enabled comparison of VO₂max across periods with and without exposure to tamsulosin and tadalafil, revealing a less-reported effect of a common medication.

## Conclusions

We present a case of a competitive endurance athlete who demonstrated a decline in estimated VO₂max temporally associated with daily tamsulosin therapy for LUTS, with partial recovery after transition to tadalafil. These findings highlight a potential, underrecognized impact of α₁-adrenergic blockade on aerobic performance and exercise capacity in the context of a routinely prescribed medication. Further studies using larger cohorts and controlled physiologic parameters are warranted.
